# Implementation of a comprehensive intervention for patients at high risk of cardiovascular disease in rural China: A pragmatic cluster randomized controlled trial

**DOI:** 10.1371/journal.pone.0183169

**Published:** 2017-08-16

**Authors:** Xiaolin Wei, John D. Walley, Zhitong Zhang, Guanyang Zou, Weiwei Gong, Simin Deng, Anthony D. Harries, Joseph P. Hicks, Marc K. C. Chong, James N. Newell, Jieming Zhong, Min Yu

**Affiliations:** 1 Division of Clinical Public Health and Institute of Health Policy Management and Evaluation, Dalla Lana School of Public Health, University of Toronto, Toronto, Canada; 2 Nuffield Centre for International Health and Development, University of Leeds, Leeds, United Kingdom; 3 China Global Health Research and Development, Shenzhen, China; 4 Zhejiang Centre for Disease Control and Prevention, Hangzhou, China; 5 International Union against Tuberculosis and Lung Disease, Paris, France and London School of Hygiene and Tropical Medicine, London, United Kingdom; 6 School of Public Health and Primary Care, Chinese University of Hong Kong, Hong Kong, China; UMCU, NETHERLANDS

## Abstract

**Objective:**

This study aims to assess whether a standard intervention package of cardiovascular disease (CVD) care was being delivered effectively, and if it was associated with improved lifestyle and biomedical indicators.

**Methods:**

In rural China, we implemented a pragmatic cluster randomized controlled trial for 12 months, randomized at the township hospital level, and compared with usual care. Intervention case management guideline, training and performance monitoring meeting and patient support activities were designed to fit within the job description of family doctors in the township hospitals and comprised: 1) prescription of a standardised package of medicines targeted at those with hypertension or diabetes; 2) advice about specific lifestyle interventions; and 3) advice about medication adherence. Participants were 50–74 years old, had hypertension *and* CVD risk scores >20% *or* diabetes, but were excluded if a history of severe CVD events. We also randomly selected 100 participants from six selected clusters per arm as a panel to collect intermediate biomedical indicators over time.

**Results:**

A total of 28,130 participants, in 33 intervention and 34 control township hospitals, were recruited. Compared with the control arm, participants in the intervention arm had substantially improved prescribing rates of anti-hypertensives, statins and aspirin (P<0.001), and had higher medication taking rates of aspirin and statins (P<0.001). Mean systolic and diastolic blood pressures were similar across both arms (0.15 mmHg, P = 0.79, and 0.52 mmHg, P = 0.05, respectively). In the panel, (950) rates of smoking (OR = 0.23, P = 0.02) and salt intake (OR = 2.85, P = 0.03) were significantly reduced in the intervention versus control arms, but there were no statistically significant improvement over the 12 month follow-up period in biomedical indicators (P>0.05).

**Conclusion:**

Implementation of the package by family doctors was feasible and improved prescribing and some lifestyle changes. Additional measures such as reducing medication costs and patient education are required.

**Trial registration:**

Current Controlled Trials ISRCTN58988083

## Introduction

Cardiovascular disease (CVD) is the leading cause of morbidity and mortality worldwide. Primary prevention of CVD requires adequately controlled blood pressure, diabetes mellitus and hyperlipidaemia as well as a reduction in obesity, smoking and alcohol consumption [1]. Hypertension is responsible for over 50% of strokes and 25% of CVD worldwide [2]. The global burden of diabetes is no less challenging with over 400 million people estimated to have the disease, half of whom are undiagnosed, with complications being a major cause of disability and reduced quality of life [3]. Every year over nine million people die from hypertension-related disease and nearly five million die from diabetes, with most of this mortality being premature [4]. Despite this huge disease burden, access to prevention and treatment remains out of reach for most people in low- and middle-income countries [5].

In China, CVD accounts for 38% of total mortality [6]. In 2010, the country had an estimated 266 million people with hypertension, 180 million with hyperlipidaemia, 92 million with diabetes and 240 million who were overweight [7]. As a result of these risk factors CVD events are predicted to increase by 23% from 2010 to 2030, resulting in 21.3 million additional CVD events and 7.7 million deaths [8]. Despite this heavy burden of disease the health services generally fail to detect and manage hypertension, diabetes and other CVD risk factors effectively. Surveys in China demonstrate that only 24% of hypertensive patients knew their condition, only 19% were on therapy and less than 5% had their blood pressure adequately controlled [9]. Adherence to medication is a major issue as less than 20% of hypertensive patients took anti-hypertensive medication in a timely way [10]. Similarly, a national survey in 2010 showed a Type II diabetes prevalence of 12% among adults, with only 26% receiving treatment [11].

CVD risk reduction cannot be achieved by treating hypertension or diabetes as separate diseases [12] [13]. A more comprehensive approach is needed. Patients with a calculated 10-year CVD risk (i.e. risk of coronary heart disease or stroke) of 20% or above are in need of therapeutic and lifestyle interventions delivered predominantly at the primary care level [14]. Studies have shown that medications such as modern anti-hypertensives, statins, and aspirin, especially in low-dose combinations, can substantially reduce CVD events [15, 16]. Healthy lifestyle interventions may have moderate effects in CVD risk reduction [1]. Smoking cessation and increased exercise are important in reducing long-term morbidity and mortality [17, 18]. Salt reduction or substitution can lead to reduced blood pressure [19], with a study in China showing that salt substitution reduced mean systolic blood pressure by 3.7 mmHg [20].

In 2009 China embarked on a series of health reforms aiming to build a primary care oriented system. Key components include the provision of a universal health insurance package and basic public health services, including detection and management of hypertension and diabetes at the community level. In rural areas, the township hospital is responsible for providing clinical care to the resident population, including public health activities such as the follow-up of patients according to national hypertension and diabetes guidelines [10]. However, the management of hypertension and diabetes is not integrated, and there is no specific medication adherence support or systematic health education [21]. After developing a suitable intervention of a comprehensive approach, we initiated a pragmatic, cluster-randomized controlled trial (cRCT) in the primary care setting of rural China to assess the health effects of the interventions over two years. Here we aimed to assess whether these interventions of the trial were being delivered over a 12-month period, were associated with reduced blood pressure, and, in a smaller sub-group, whether there were any differences in a range of intermediate outcomes at 12 months.

## Materials and methods

### Ethics statement

This study was approved by the Ethics Review Board of the University of Leeds, UK (reference HSLTLM/12/010) and the Ethics Committee of Zhejiang Provincial Centre for Disease Control and Prevention, China (reference 18/06/2012). All participants have given written informed consent before participating in the study.

### Study design and participants

This study was based on an on-going, two-arm, parallel-group, cluster-randomized controlled trial that aims to answer to what extent the intervention strategies have been implemented. This was designed as a pragmatic trial as i) eligibility criteria for hospitals and patients were broad and inclusive to represent typical rural populations; ii) medications were not freely provided but were covered up to 30% by the rural health insurance scheme; and iii) delivery of the services was embedded within the routine job descriptions of family doctors. To assess delivery of interventions we compared the following outcomes between intervention and control townships: i) the prescribing and taking of medications at quarterly intervals over 12 months; and ii) blood pressure readings at quarterly intervals over 12 months. To assess intermediate outcomes at 12 months we selected a sub-group panel in which we measured medication adherence using Morisky questionnaire, healthy lifestyles, changes in body mass index (BMI), glycated hemoglobin levels (HbA_1c_) and lipid profiles at 12 months compared with those found at baseline (S1 Table).

Three counties located in central Zhejiang province were selected on the basis that their townships hospitals had electronic health records and agreed to participate in the trial [22]. We excluded one township whose hospital was used for a pilot study. In each township we recruited patients aged 50 to 74 years who held permanent residence in the township and were either hypertensive with a 10-year CVD risk of 20% or higher calculated using the Asian Equation [23], based on information available in the electronic health records [10], or had a recorded medical history of Type 2 diabetes mellitus. All eligible participants were identified from existing health records. We excluded patients with mental health problems, physical disabilities, history of severe CVD events, or other severe diseases. We also excluded patients who were hospitalized during recruitment; patients who had serious adverse effects to the recommended drugs; those whose diastolic blood pressure were lower than 60 mmHg; or those who had high risk of CVD but did not have hypertension or diabetes; or those who declined to participate in the trial. Potentially eligible participants were asked to visit the township hospital where they were assessed by family doctors for eligibility criteria, consulted about their willingness to participate in the study and invited to provide informed written consent. Patients in Zhejiang were used to visit their own township hospitals based on health insurance regulations, also due to the familiarity of doctors, thus the potential for cross contamination is low [24]. Participant recruitment lasted from December 2013 to May 2014, and the participants had been followed up for 12 months when this analysis was conducted. The authors confirm that all ongoing and related trials for this intervention are registered (S1 File).

### Sample size

The sample size for the whole study group was based on the primary outcome measure: the CVD event rate. We assumed the intervention would lead to a relative reduction of at least 20% from the current CVD event rate of 5% within two years, based on published values. We estimated to require 32 clusters per arm with 450 participants per cluster to detect such a difference with 90% power, assuming a coefficient of variation of 0.15 and testing at the 5% level.

The sample size calculation for the panel sub-group was conducted to power the detection of effects in outcomes measured from the panel patients’ blood samples: HbA_1c_, total serum cholesterol (TC) and low-density lipoprotein (LDL) levels. This sample size was based on detecting changes in TC, because it was expected to display the smallest change. Based on pilot data and values from the literature, and assuming just one-third of patients took their statins, we expected a 6% relative reduction in the level of TC. We estimated we required 6 clusters per arm to detect this change with 90% power, with 100 participants per cluster and 10% loss to follow-up, assuming an intracluster correlation coefficient of 0.01, and testing at the 5% level. Full details of the sample size calculations are given in the trial protocol [22].

### Randomization and masking

An independent biostatistician randomly allocated eligible township hospitals to intervention or control arms in a 33:34 ratio using sequential numbers without stratification. Allocation took place after subject recruitment. All eligible participants in each township received the study treatment allocated to their township hospital. Blinding was not feasible for patients or providers. Written informed consent was obtained from both hospital directors and individuals. For the panel, six clusters (100 participants per cluster) were randomly selected from each arm.

### Intervention arm

The intervention case management guideline, training and performance monitoring meeting and patient support activities were designed to fit within the job description of family doctors in the township hospitals. These comprised: 1) prescription of a standardised package of highly effective medicines targeted at those with hypertension or with diabetes; 2) advice about specific lifestyle interventions; and 3) advice about medication adherence [22]. Intervention activities were documented in a deskguide, which included a clinical treatment algorithm and information on the use of recommended medicines (on the essential medicine list); healthy lifestyle education; medication adherence support; and how to change and replace existing medicines if necessary [22]. Training of trainers on the use of the deskguide was provided for senior doctors on a half-day course annually. These doctors then became trainers in their own township hospitals and conducted initial and refresher trainings quarterly. In addition, the township hospitals discussed the performance and improvement strategies in their monthly internal meetings based on the feedback of performance indicators sent by local CDCs.

All hypertensive patients in this trial had high risk of CVD, thus the clinical treatment algorithm recommended, for all hypertensive patients in the trial, prescribing a standard combination [25] of two anti-hypertensives (two different kinds selected from thiazide-diuretics, calcium-channel blockers, angiotensin-converting enzyme inhibitors, angiotensin II receptor blockers, or beta-blockers, according to the Chinese Hypertension Guideline) [26], a statin, and a low dose of aspirin. Patients with diabetes only were prescribed a standard combination of one anti-hypertensive, a statin, and a low dose of aspirin, plus their anti-diabetic medicines if any. Patients already on other anti-hypertensive medicines were advised to switch to the modern medicines in the standardised packages. All doctors were educated of side effects and contradictions of the medicines, such as not giving aspirin to haemorrhagic patients, and how to detect the early sign of any side effects. According to the health insurance policies, patients could purchase their medications from the hospital pharmacy or any qualified street pharmacies using doctor prescriptions according to health insurance policies. Doctors were trained to recommend the medications that were prescribed but not actually taken in the following consultations.

Participants were followed up by their family doctor monthly in the township hospitals or during the doctor’s home visit, and were reminded of follow-up appointment through phone call/SMS. The consultation included health education, focusing on smoking cessation, healthy eating, salt reduction, and reduction in alcohol consumption. Patients were assisted to select a family treatment supporter who received advice on how to provide support at home to improve medication and lifestyle change adherence (S1 Text).

### Control arm

In the control arm, management of hypertension and diabetes continued conventional clinical consultations, with treatment provided according to the individual family doctor’s existing knowledge and discretion (including of current national guidelines if aware of them). No specific medication adherence support was provided, and health education, if any, was non-systematic.

### Data collection

We aimed to follow up all patients at least once per quarter, with follow up data collected during all consultations. During consultations doctors recorded patient prescriptions and their actual intake of medications. They also recorded patients’ blood pressures, measured using mercury sphygmomanometers after patients had sat for five minutes. In both arms doctors entered data on patients’ blood pressure, height and weight into the routine internet-based public health management information system. Additionally, those participants selected for the panel population underwent an examination, where doctors recorded their BMI, blood pressure, and levels of HbA_1c_, TC and LDL at baseline, 12 and 24 months after randomization [22]. All laboratory specimens and tests results were quality assured at the provincial central reference laboratory. Patients were also interviewed regarding their smoking status, alcohol consumption, physical exercise, and drug adherence. This data was recorded in a parallel internet-based trial management system designed for family doctors.

### Outcomes

The primary outcome of the trial is the incidence of severe CVD events (i.e., coronary heart disease and stroke) over 36 months of follow-up recorded by Zhejiang’s CVD surveillance system, which will not be reported in this process evaluation paper. In this study we report descriptively quarterly outcomes for rates of prescribing and actual drug-taking of recommended highly effective medicines, and mean systolic and diastolic blood pressure (mmHg), for all participants. We also analyze changes in these outcomes between baseline and 12 month follow-up. Similarly, in the panel population we report and analyze changes in patient intermediate outcomes at baseline and 12 months for levels of HbA_1c_ (%), TC (mmol/L) and LDL (mmol/L), as well as rates of smoking and alcohol consumption, salt intake, exercise, BMI, and participant adherence to medications (i.e., whether taking medicines timely and with full dosage among patients who have taken medicines) using the Morisky scale, where achieving 6–8 within a scale of 0–8 was regarded as good adherence [27]. Control of hypertension was defined as both a systolic pressure ≤ 140 mmHg and a diastolic pressure ≤ 90 mmHg. Other outcomes listed in the protocol will be reported in a qualitative process evaluation paper to be published elsewhere.

### Statistical analysis

All data from the internet-based management system were exported to SPSS® 20.0 (Chicago, USA) and analyzed using SAS® 9.4 (Cary NC, USA). Data were analyzed according to the randomized allocation of their township, and irrespective of their adherence to the protocol. Another biostatistician (MKCC) who was responsible for data analysis was blinded to the treatment allocation. Descriptive analyses of means, medians and proportions were used to report baseline socio-demographic and clinical information. For the panel sub-group, the intervention effects on continuous 12 month outcomes (i.e. systolic and diastolic blood pressure, HbA1_c_, TC, LDL and BMI) were analyzed using linear mixed-effect models adjusted for clustering (i.e. a random effect of township hospital), and baseline covariates, namely age, gender, education levels, and annual incomes. The intervention effects on binary 12 month outcomes, such as changes in smoking rates, were analyzed using mixed-effects logistic regression models adjusting for clustering and the same demographic/socio-economic factors. The effects of the intervention on 12 month systolic and diastolic blood pressures in the whole population group were analyzed using linear mixed-effect models adjusting for clustering, and other potentially confounding covariates as above. Hypothesis testing was two-sided and at the 5% level. Cases with missing outcome or covariate data were excluded. Standard model assumptions, such as homoscedasticity and normality of errors/random effects, were assessed to ensure models were valid.

## Results

### Delivery of interventions for the whole study group

**Fig 1:**
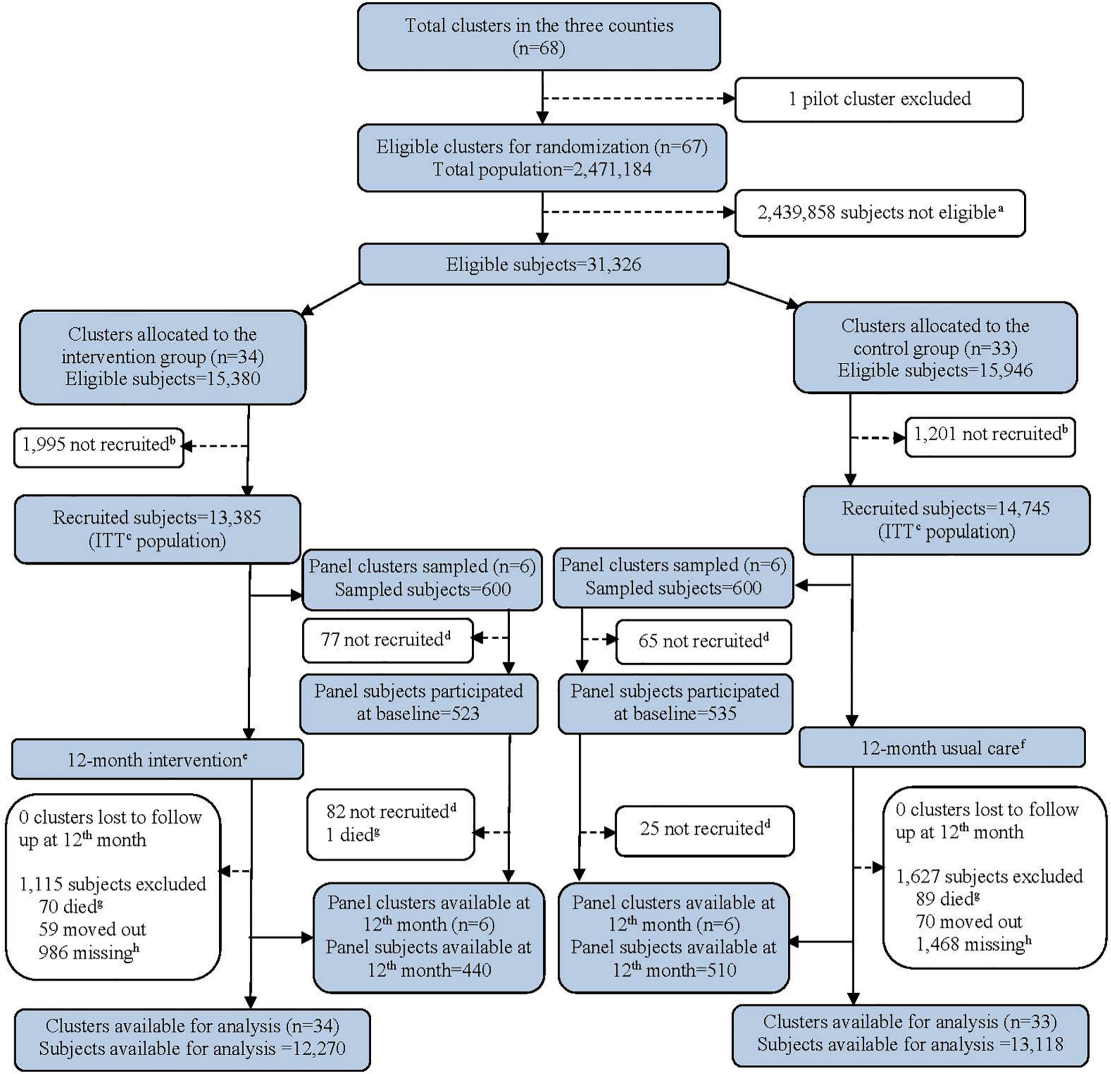
Trial profile. a. Eligible subjects included those 1) aged 50–74; 2) with a calculated 10-year CVD risk≥20% and having hypertension; or 3) diagnosed as diabetic. Subjects excluded were those who had 1) mental diseases; 2) cancers; 3) acute coronary heart disease or had previously suffered stroke; 4) diastolic blood pressure <60 mmHg; or those who were 5) out of town for more than 3 months or non-locals; 6) urban residents; or those who 7) couldn’t take recommended medicines due to other severe diseases such as cancer. b. Subjects who did not sign consent forms were not recruited. c. Subjects did not participant in the survey and data collection despite being reminded 3 times within the last 2 weeks. d. Intervention package includes: 1) healthy lifestyle education; 2) drug therapies; 3) adherence support. e. Usual care refers to health care following routine procedures at the discretion of individual family doctors. f. Subjects died from reasons other than coronary heart disease or stroke during the 12 months were excluded. g. Missing cases: those who had been absent in a consecutive of 6 months or two follow-up appointments at quarterly basis.

All the 67 eligible township hospitals are currently participating in the study. A total of 28,130 hypertensive or diabetes patients met the inclusion criteria and were enrolled between December 2013 and May 2014. Demographic and clinical characteristics at baseline are shown in Table 1. In each arm, the majority of patients were 60 years or older, married, had an education level of primary school or lower, and had an average per capita annual income of RMB 12,900 (USD 2,150). In each arm, around two thirds of patients had hypertension (with or without diabetes) and one third had diabetes alone. There were no substantial imbalances in characteristics between arms at baseline. At 12 months, all 67 clusters were successfully followed up, with 12,270 (92%) in the intervention arm and 13,118 (90%) in the control arm successfully followed up and included in the analysis. Loss to follow-up was similar between the intervention and control arms (Fig 1).

**Table 1:** Demographic, socioeconomic and disease characteristics of patients recruited in the trial.

	Intervention group N or mean (% or SD)	Control group N or mean (% or SD)	Total N or mean (% or SD)
Number of patients	13385	14745	28130
Age	64.3 (6.3)	64.3 (6.1)	64.3 (6.2)
Gender			
Male	6443 (48.1)	7316 (49.6)	13759 (48.9)
Female	6942 (51.9)	7429 (50.4)	14371 (51.1)
Married^1^	11618 (98.1)	12763 (98.4)	24381 (98.3)
Education^1^			
Primary (≤6 years)	10356 (77.6)	11070 (75.4)	21426 (76.5)
High school (7–12 years)	2691 (20.2)	3234 (22.0)	5925 (21.1)
College and above (≥12 years)	290 (2.2)	382 (2.6)	672 (2.4)
Annual household income (RMB)	35,300 (40,400)	39,800 (51,000)	37,700 (46,500)
Annual per capita income (RMB)	12,000 (11,800)	13,700 (19,500)	12,900 (16,400)
Hypertension and diabetes diagnoses^1^			
Including hypertension	8781 (65.7)	9087 (62.2)	17868 (63.9)
Only diabetes	4585 (34.3)	5521 (37.8)	10106 (36.1)

SD: Standard Deviation, RMB: Renminbi at the rate of 1USD = 6RMB in 2013/14

^1^ Missing values were excluded for proportion calculations: 1542 (11.5%) and 1774 (12.0%) missing values in intervention and control group for marriage status; 48 (0.4%) and 59 (0.4%) missing values in intervention and control group for education; 19 (0.1%) and 137 (0.9%) missing values in intervention and control group for hypertension and diabetes diagnoses.

### Prescribing and taking of medications

**Fig 2:**
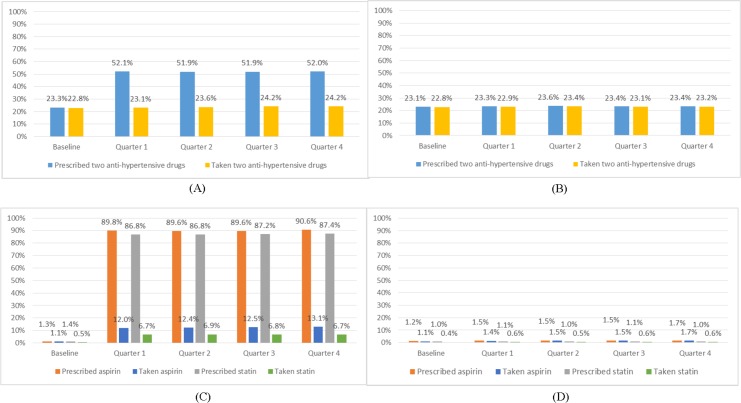
Proportions of prescribing and taking of medicines among recruited hypertensive patients*. (A) Proportion of patients having two anti-hypertensive drugs prescribed and taken in the intervention group; (B) Proportion of patients having two anti-hypertensive drugs prescribed and taken in the control group; (C) Proportion of patients having aspirin and statin prescribed and taken in the intervention group; (D) Proportion of patients having aspirin and statin prescribed and taken in the control group. * At 12 months follow-up (quarter 4) compared to the control arm in the intervention arm a significantly higher proportion of patients had been prescribed (OR = 3.55, 95% CI: 3.31 to 3.80, P<0.001), but had not taken (OR = 1.06, 95% CI: 0.98 to 1.14, P = 0.15), two antihypertensives; a significantly higher proportion of patients had been prescribed (OR = 565.77, 95% CI: 469.12 to 682.33, P<0.001) and had taken (OR = 8.85, 95% CI: 7.37 to 10.63, P<0.001), aspirin; and a significantly higher proportion of patients had been prescribed (OR = 656.24, 95% CI: 523.36 to 822.85, P<0.001) and had taken (OR = 11.79, 95% CI: 8.76 to 15.87, P<0.001) statins. Comparisons based on multivariate logistic mixed-effects models, adjusted for cluster effects, baseline measurement and demographic/socioeconomic factors.

Results for all hypertensive patients are given in Fig 2A–2D. Prescription of two anti-hypertensive drugs at baseline was low, at around 23% in each arm. At 12 months (quarter 4) follow-up, around 50% of patients were prescribed two anti-hypertensives in the intervention arm compared with 20% in the control arm (OR = 3.55, 95% CI: 3.31 to 3.80, P<0.001). Similarly, aspirin and statin prescription and taking rates were very low at baseline, at less than 1.1% in each arm. At 12 months, around 91% were prescribed aspirin and 87% prescribed statins in the intervention arm compared with around 1.7% and 1.0% respectively in the control arm (OR = 565.77, 95% CI: 469.12 to 682.33, P<0.001 and OR = 656.24, 95% CI: 523.36 to 822.85, P<0.001, respectively). At 12 months, the drug-taking rates of two anti-hypertensives were similar for intervention and control arms at around 24% (OR = 1.06, 95% CI: 0.98 to 1.14, P = 0.151), while drug-taking rates of aspirin and statin were much higher in the intervention arm (13% and 7% respectively) than in the control arm (1.7% and 0.6% respectively) (OR = 8.85, 95% CI: 7.37 to 10.63, P<0.001 and OR = 11.79, 95% CI: 8.76 to 15.87, P<0.001).

**Fig 3:**
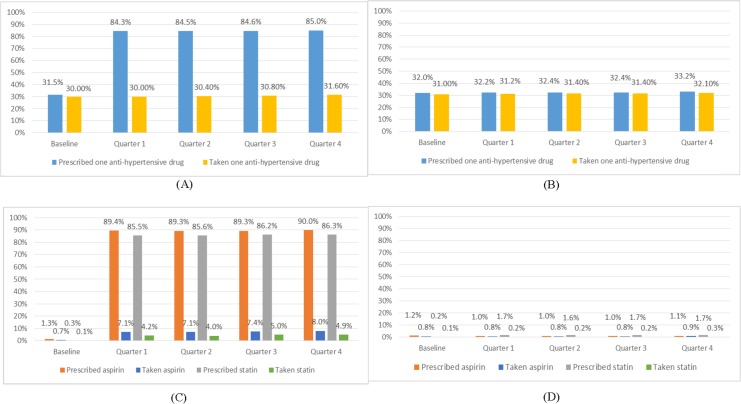
Proportions of prescribing and taking of medicines among recruited patients with diabetes only*. (A) Proportion of patients having one anti-hypertensive drug prescribed and taken in the intervention group; (B) Proportion of patients having one anti-hypertensive drug prescribed and taken in the control group; (C) Proportion of patients having aspirin and statin prescribed and taken in the intervention group; (D) Proportion of patients having aspirin and statin prescribed and taken in the control group. * At 12 months follow-up (quarter 4) compared to the control arm in the intervention arm a significantly higher proportion of patients had been prescribed (OR = 11.41,95% CI:10.24 to 12.71, P<0.001), but had not taken (OR = 0.98, 95% CI: 0.89 to 1.07, P = 0.60), one anti-hypertensive; a significantly higher proportion of patients had been prescribed (OR = 806.98, 95% CI: 597.55 to 1089.79, P<0.001) and had taken (OR = 9.53, 95% CI: 6.84 to 13.28, P<0.001) aspirin; and a significantly higher proportion of patients had been prescribed (OR = 360.86, 95% CI: 282.59 to 460.81, P<0.001) and had taken (OR = 18.93, 95% CI: 10.55 to 33.97, P<0.001) statins. Comparisons based on multivariate logistic mixed-effects models, adjusted for cluster effects, baseline measurement and demographic/socioeconomic factors.

Results for patients with diabetes only are given in Fig 3A–3D. As with anti-hypertensive drugs, prescription rates for aspirin and statins were similar between the two arms at baseline. At 12 months, around 85% of patients were prescribed an anti-hypertensive in the intervention arm compared with 33% in the control arm (OR = 11.41, 95% CI: 10.24 to 12.71, P<0.001), and around 90% were prescribed aspirin and 86% prescribed a statin in the intervention arm compared with around 1.1% and 1.7% respectively in the control arm (OR = 806.98, 95% CI: 597.55 to 1089.79, P<0.001 and OR = 360.86, 95% CI: 282.59 to 460.81, P<0.001). At 12 months, drug-taking rates of one anti-hypertensive were similar for intervention and control arms, at around 32% (OR = 0.98, 95% CI: 0.89 to 1.07, P = 0.596), while drug-taking rates of aspirin and a statin were higher in the intervention arm (8% and 5% respectively) than in the control arm (0.9% and 0.3% respectively) (OR = 9.53, 95% CI: 6.84 to 13.28, P<0.001 and OR = 18.93, 95% CI: 10.55 to 33.97, P<0.001).

Adding all participants together, 72% took at least one antihypertensive medicines. After 12 months, 66% and 65% respectively took medicines containing at least one modern anti-hypertensive drug in the intervention arm and control arm. In addition, 18% and 16% of participants in the intervention and control arm respectively did not take modern drugs but took herbal medicines.

### Blood pressure

**Fig 4:**
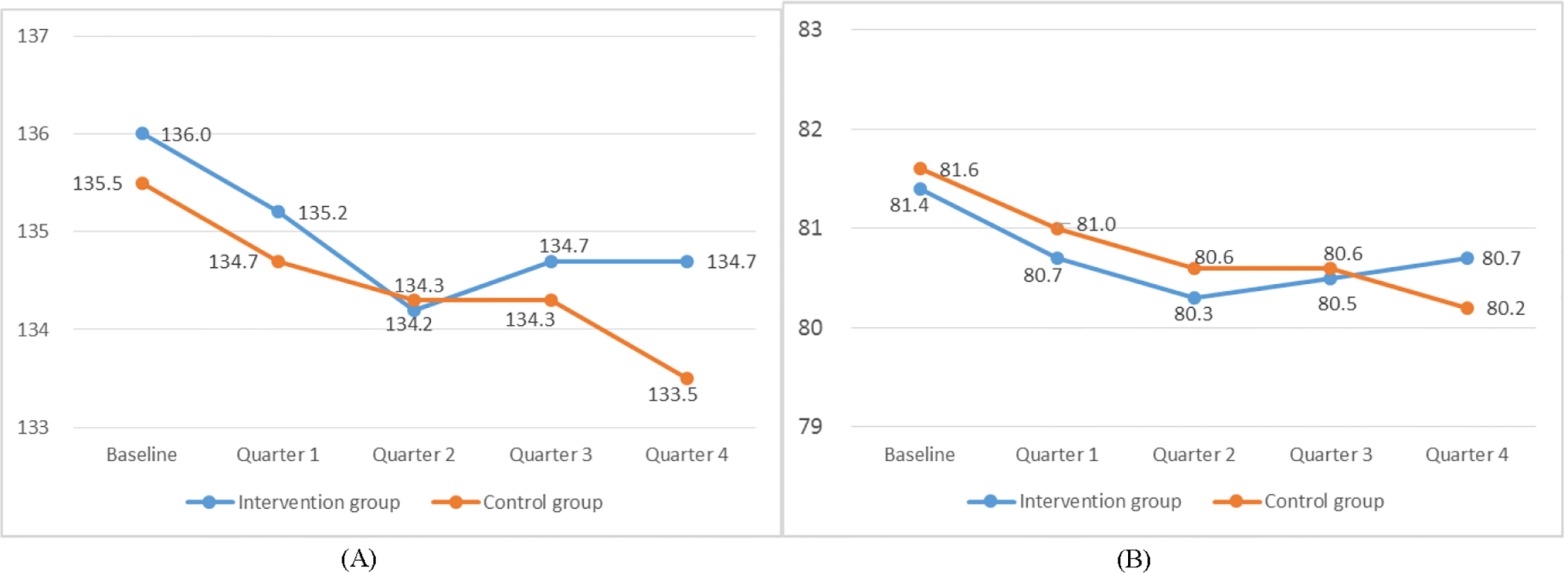
Blood pressure changes (mmHg) by quarter for patients recruited in the trial*. (A) Quarterly changes in mean systolic blood pressure in the intervention and the control group; (B) Quarterly changes in mean diastolic blood pressure in the intervention and the control group. *No significant differences between intervention and control arms by 12 months follow-up (quarter 4) in terms of either systolic blood pressure (0.15, 95% CI: -0.96 to 1.26, P = 0.79, ICC = 0.07) or diastolic blood pressure (0.52, 95% CI: -0.02 to 1.01, P = 0.05, ICC = 0.04). Comparisons based on multivariate linear mixed-effects models adjusted for cluster effects, baseline measurements and demographic/socioeconomic factors.

Mean systolic and diastolic blood pressures in the intervention and control arms for all patients (hypertensive and diabetic) combined at quarterly intervals during the initial 12 months are shown in Fig 4A and 4B). Mean systolic pressure declined from around 136 to 134 mmHg and mean diastolic pressure from around 81 to 80 mmHg in both intervention and control arms, but there were no significant differences between arms after adjusting for cluster effects and demographic factors (0.15, 95% CI -0.96 to 1.26, P = 0.79, and 0.52, 95% CI -0.02 to 1.01, P = 0.05, respectively). At baseline, the blood pressure control rate was 75% among all patients. For antihypertensive patients, blood pressure was controlled in 60% of hypertensive patients in the intervention arm and 64% in the control arm at baseline, rising to 75% and 78% respectively at 12 months (S1 Fig). However, these differences were not statistically significant at 12 months (OR = 1.07, 95% CI: 0.73 to 1.57, P = 0.74).

### Intermediate outcomes at 12 months in the panel sub-group

#### Baseline characteristics

Demographic and clinical characteristics of the 1,058 patients in intervention and control clusters who participated in the panel sample are shown in Table 2. The panel population were relatively less likely to be married (88.1% in the panel compared to 98.3% in the main trial population), relatively poorer (per capita annual income RMB 9,791/ USD 1,632 in the panel compared to RMB 12,900 / USD 2,150 in the main trial population), and a higher proportion of the panel population had hypertension (74.7%) compared with that of the general participants (63.9%). However, there were no substantial imbalances regarding key clinical characteristics between arms in the panel population. At 12 months, 440 (84%) in the intervention clusters and 510 (95%) in the control clusters were successfully followed up and included in the analysis (Fig 1).

**Table 2:** Demographic, socioeconomic and disease characteristics of patients recruited in the panel.

	Intervention group N or mean (% or SD)	Control group N or mean (% or SD)	Total N or mean (% or SD)
Number of patients	523	535	1058
Age	65.4 (6.6)	65.8 (6.4)	65.6 (6.5)
Gender			
Male	242 (46.3)	237 (44.3)	479 (45.3)
Female	281 (53.7)	298 (55.7)	579 (54.7)
Married	456 (87.2)	476 (89.0)	932 (88.1)
Education			
Primary (≤6 years)	419 (80.1)	402 (75.1)	821 (77.6)
High school (7–12 years)	92 (17.6)	116 (21.7)	208 (19.7)
College and above (≥12 years)	12 (2.3)	17 (3.2)	29 (2.7)
Annual household income (RMB)	29,009 (62,418)	33,540 (43,625)	31,303 (53,753)
Annual per capita income (RMB)	9,069 (14,956)	10,496 (13,965)	9,791 (14,474)
Hypertension and diabetes diagnoses			
Including hypertension	387 (74.0)	403 (75.3)	790 (74.7)
Only diabetes	136 (26.0)	132 (24.7)	268 (25.3)

SD: Standard Deviation, RMB: Renminbi at the rate of 1USD = 6RMB in 2013/14

### Drug adherence, lifestyle and changes in intermediate outcomes

At 12 months, good drug adherence was reported by 66% of participants in the intervention arm, compared with 47% for the control arm (P<0.001, OR = 2.23, 95% CI: 1.701 to 2.932). Smoking rates were significantly reduced in the intervention arm (4% reduction absolute. Throughout this section changes are given in absolute terms) compared with the control arm (2.5% increase) (OR = 0.23, 95% CI: 0.07 to 0.78, P = 0.02, see Table 3). In addition, more patients in the intervention arm took less salt than the control arm (50% vs. 34%, OR = 2.85, 95% CI: 1.12 to 7.27, P = 0.03). Patients in the intervention arm reported drinking less alcohol and had more exercise (32% vs. 15%, and 31% and 18%, respectively), but these changes were not statistically significant (P = 0.21 and P = 0.23). Changes in BMI, systolic and diastolic blood pressure, hemoglobin levels, TC and LDL profiles between baseline and 12 months for all patients and those with hypertension or diabetes in intervention and control arms are shown in Table 4. There were not statistically significant, apart from a statistically significantly greater 12 month-baseline decrease in BMI for patients with diabetes only compared in the intervention arm compared to the control arm (-0.81, 95% CI: -1.49 to -0.13, P = 0.02).

**Table 3:** Lifestyle changes of patients in the panel.

	All patients, N (%)		Multivariate analysis		Patients with hypertension*, N (%)		Multivariate analysis		Patients with diabetes only, N (%)		Multivariate analysis	
	Intervention	Control	Odds ratio (95% CI)^1^	p-value^2^	Intervention	Control	Odds ratio (95% CI)^1^	p-value^2^	Intervention	Control	Odds ratio (95% CI)^1^	p-value^2^
No. of patients	440	510			324	385			116	125		
Smoking												
Baseline	81 (18.4)	88 (17.3)			71 (21.9)	69 (17.9)			10 (8.6)	19 (15.2)		
12th month	64 (14.5)	101 (19.8)	0.23 (0.07, 0.78)	0.02	53 (16.4)	81 (21.0)	0.21 (0.06, 0.73)	0.01	11 (9.5)	20 (16.0)	0.91 (0.09, 11.5)	0.95
Tried to quit during last 12 months	21 (32.8)	20 (19.8)			16 (30.2)	14 (17.3)			5 (45.5)	6 (30.0)		
Alcohol drinking												
Baseline^3^	98 (22.3)	119 (23.3)			80 (24.8)	88 (22.9)			18 (15.5)	31 (24.8)		
12th month	87 (19.8)	131 (25.7)	0.50 (0.17, 1.49)	0.21	68 (21.0)	99 (25.7)	0.50 (0.16, 1.52)	0.21	19 (16.4)	32 (25.6)	0.78 (0.13, 4.76)	0.79
Tried to quit during last 12 months^4^	28 (32.2)	15 (12.1)			20 (29.4)	10 (10.4)			8 (42.1)	5 (17.9)		
Salt intake compared with baseline												
More or no change	220 (50.0)	336 (65.9)			165 (50.9)	247 (64.1)			55 (47.4)	89 (71.2)		
Less	220 (50.0)	174 (34.1)	2.85 (1.12, 7.27)	0.03	159 (49.1)	138 (35.8)	2.29 (0.83, 6.33)	0.11	61 (52.6)	36 (28.8)	3.32 (0.90, 12.2)	0.07
Exercise compared with baseline												
More	135 (30.7)	92 (18.0)			105 (32.4)	76 (19.7)			30 (25.9)	16 (12.8)		
Less or no change	305 (69.3)	318 (82.0)	1.90 (0.66, 5.46)	0.23	219 (67.6)	309 (80.3)	1.76 (0.68, 4.52)	0.24,	86 (74.1)	109 (87.2)	3.40 (0.54, 21.4)	0.19

^1^ Odds ratios comparing intervention to control arm outcomes, their 95% CIs and

^2^ p-values obtained from multivariate logistic mixed-effects models comparing intervention and control groups, adjusted for cluster effects, baseline measurements and demographic/socioeconomic factors

^3^ Missing values were excluded for proportion calculation: 1 (0.2%) missing value in intervention group for all patients; 1 (0.3%) missing value in intervention group for patients with hypertension

^4^ Missing values were excluded for proportion calculation: 7 (5.3%) missing values in control group for all patients; 3 (3.0%) missing values in control group for patients with hypertension; 4 (12.5%) missing values in control group for patients with only diabetes.

*Patients with hypertension include patients with only hypertension and patients with both hypertension and diabetes.

**Table 4:** Changes of metabolic and biomedical indicators of patients in the panel.

	All patients, Mean (SD)		Multivariate analysis		Patients with hypertension*, Mean (SD)		Multivariate analysis		Patients with diabetes only, Mean (SD)		Multivariate analysis	
	Intervention	Control	Mean difference (95% CI)^1^	p-value^2^	Intervention	Control	Mean difference (95% CI)^1^	p-value^2^	Intervention	Control	Mean difference (95% CI)^1^	p-value^2^
No. of patients	440	510			324	385			116	125		
BMI												
Baseline	24.8 (3.2)	24.0 (3.5)			24.9 (3.2)	24.1 (3.6)			24.6 (3.1)	23.5 (3)		
12th month	24.1 (3.2)	23.8 (3.4)			24.3 (3.3)	23.9 (3.5)			23.8 (3.1)	23.6 (3.1)		
Difference	-0.7 (2.1)	-0.1 (2.7)	0.22 (-0.78, 0.08)	0.11	-0.6 (2.2)	-0.2 (2.7)	-0.16 (-0.60, 0.29)	0.49	-0.7 (2.1)	0.4 (2.4)	-0.81 (-1.49, -0.13)	0.02
SBP (mmHg)												
Baseline	142.6 (17.5)	140.8 (19.1)			145.1 (17.4)	143.1 (19.4)			135.4 (15.8)	133.6 (16.2)		
12th month	141.7 (17.3)	13.5 (17.1)			143.6 (17.8)	140.9 (17.2)			136.3 (14.4)	135.1 (15.8)		
Difference	-0.9 (22.5)	-1.3 (23.3)	-0.73 (-6.80, 5.34)	0.86	-1.5 (23.1)	-2.2 (23.2)	0.70 (-6.39, 7.78)	0.84	0.9 (20.8)	1.5 (21.4)	-0.01 (-6.99, 6.97)	0.97
DBP (mmHg)												
Baseline	85.2 (9.4)	84.8 (10.5)			86.7 (9.6)	85.9 (10.7)			81 (7.4)	81.5 (9.2)		
12th month	81.3 (10.4)	82.5 (9.2)			81.9 (10.5)	83 (9.2)			79.6 (9.9)	81 (9.1)		
Difference	-3.9 (12.9)	-2.2 (12.8)	-1.40 (-4.08, 1.29)	0.31	-4.9 (13.2)	-2.8 (13.1)	-1.21 (-4.18, 1.76)	0.43	-1.4 (11.7)	-0.5 (11.7)	-1.33 (-4.78, 2.11)	0.44
HbA1c (%)												
Baseline	6.8 (2.1)	6.5 (2.1)			6.40 (2.1)	6.10 (1.9)			7.10 (2.2)	7.70 (2.4)		
12th month	6.7 (2.1)	7.3 (5.1)			6.60 (1.9)	7.19 (4.9)			7.06 (2.10)	7.69 (5.72)		
Difference	-0.2 (2.2)	0.8 (5.4)	-0.33 (-1.51, 0.85)	0.56	0.23 (2.2)	1.1 (5.0)	-0.42 (-1.57, 0.73)	0.45	-0.01 (2.17)	0.03 (6.27)	-0.71 (-2.43, 1.01)	0.4
Total cholesterol (mmol/L)												
Baseline	4.9 (1.0)	4.9 (1.0)			4.9 (1.0)	4.9 (1.0)			4.9 (0.9)	4.8 (0.8)		
12th month	4.6 (1.0)	4.5 (1.0)			4.5 (1.0)	4.5 (1.0)			4.7 (1)	4.6 (0.9)		
Difference	-0.4 (1.2)	-0.3 (1.2)	0.03 (-0.17, 0.22)	0.78	-0.4 (1.3)	-0.4 (1.3)	0.01 (-0.22, 0.24)	0.94	-0.2 (1)	-0.2 (1)	-0.01 (-0.23, 0.23)	0.98
LDL (mmol/L)												
Baseline	2.8 (0.8)	2.6 (0.7)			2.8 (0.8)	2.6 (0.7)			2.8 (0.7)	2.6 (0.6)		
12th month	2.6 (0.7)	2.6 (0.8)			2.6 (0.7)	2.6 (0.8)			2.7 (0.6)	2.7 (0.7)		
Difference	-0.2 (0.9)	0 (0.9)	-0.06 (-0.24, 0.12)	0.53	-0.2 (0.9)	0 (1.0)	-0.05 (-0.25, 0.15)	0.58	-0.1 (0.7)	0.1 (0.8)	-0.07 (-0.29, 0.15)	0.57

BMI = body mass index; SBP = systolic blood pressure; DBP = diastolic blood pressure; HbA_1c_ = glycosylated hemoglobin; LDL = low-density lipoprotein

^1^ Mean differences in 12 month-baseline differences between intervention and control arm outcomes, their 95% CIs and

^2^ p-values obtained from linear mixed-effect models comparing intervention and treatment groups, adjusted for cluster effects, baseline measurements and demographic/socioeconomic factors.

*Patients with hypertension include patients with only hypertension and patients with both hypertension and diabetes.

## Discussion

This is, to our knowledge, the first pragmatic randomized controlled trial that implements a comprehensive package at the primary healthcare level for high CVD risk hypertensive or diabetic patients. Though in many developed countries some of our CVD risk prevention package is now standard care, in China and most LMICs it is not generally provided. Within the framework of this trial, we conducted an analysis which showed that interventions were delivered and that these were signs of improvement at 12-months. The primary indicator (CVD events) will be analysed at the end of the trial.

A high proportion of patients in the intervention arm were given prescriptions of the anti-hypertensive drugs, a statin and aspirin, as recommended in the standard medication package [25]. Most family doctors in the intervention arm have changed prescribing behaviour by 12 months, which compares very well with rates for interventions in a systematic review [28]. However, the actual taking of medications according to these prescriptions was low. There was a modest improvement in the (previously very low) rates of prescribing statins. But the rates of taking anti-hypertensive drugs were similar between intervention and control arms. This may be because there was a relatively high level of general anti-hypertensive use at baseline in both arms (over 70%), and 75% of patients in both arms achieved targeted blood pressure control. This high drug uptake at baseline may limit the anti-hypertensive gain that we hoped for in the trial, and it was reported that patients were reluctant to switch to or add another anti-hypertensive [21]. Similar positive findings on blood pressure control were observed in urban clinics in China where patients were managed under primary care facilities [29], but these contrast with the relatively poor national average [30, 31]. In a pragmatic trial using one anti-hypertensive in a setting with low medicine usage (<7%) in China and India, only 20% of participants took the prescribed medications in 12 months, and this was associated with a small (<3 mmHg) average reduction in systolic blood pressure [32].

We did observe that a higher proportion of patients in the intervention arm took aspirin and statins compared with the control arm. However, the drug-taken rates were disappointing, around 10%, compared to that approximately 90% of patients were prescribed aspirin and statins during consultations. We felt that the gap may be that doctors, who identified the low drug-taken during consultation, were reluctant to persuade patients for a number of reasons. The worry for aspirin was related to fears of gastrointestinal bleeding [10], while the concern of statins may be economic. Statins cost over 120 RMB/ USD 20 per month, but only 30% of drug costs were covered by health insurance [21]. Patient education on these two drugs have to come from a variety of sources, including their experience of consultations with doctors from big hospitals and public educations. This is a potential area to be improved. In addition, a fixed combination pill is not available in China, though this might improve drug taking adherence compared with separate pills [16].

There were several strengths to this study. First, the intervention was developed with the provincial health authorities according to the ‘embedded research and development’ approach, where research questions are focused on local opportunities for going to scale; interventions are designed and evaluated in real-world setting with public health agencies, and the results can directly inform program development [33]. Our trial was designed to be implementable, replicable and sustainable within the routine health services, and supported government policy on essential public health interventions. The intervention package was embedded into daily primary care practice [24]. Second, the intervention package and the research procedures were tested and refined in a pilot study before the trial [21]. Third, the sample was large and patients were managed on the basis of their CVD risk rather than just treatment of their hypertension and/or diabetes.

Several limitations need to be stated. First, this was a pragmatic trial implemented by primary care staff who received half-day training annually and referesh training monthly within their own hospitals. More intensive training may be needed to target doctor-patient communication regarding the benefits of using combination medications and changing to healthier lifestyles. Second, we relied on routine reporting systems and inevitably there were missing data. Patient lifestyle indicators were self-reported. We have provided training for doctors in both arms regarding standard measurement and consultation reports [22]. Third, the study did not show any significantly improvement of biomedical indicators regarding blood pressures, BMI, HbA_1c,_ total cholesterol and LDL between baseline and 12 months in the intervention arm compared with the control arm, though there is strong evidence that the four-drug combination is associated with improvements in intermediate outcomes and a marked reduction in CVD events [15, 16]. This may be due to relatively high uptake of antihypertensive in both arms at baseline, and low uptake of aspirin and statin in the intervention arm during the intervention. To be of notice, all our medications were not supplied free-of-cost, but were prescribed in routine practice with partial reimbursement through health insurance schemes. Although countries like China have implemented universal health coverage, health insurance covers only a marginal part of outpatient costs, and this can substantially reduce the uptake of more expensive drugs such as statins. The rural health insurance needs to improve its outpatient reach to cover statins and other highly effective medications. Fourth, there was no blinding for healthcare providers or patients, although the outcome analysis was blinded. Fifth, the trial was not designed and powered to test the effects of individual components within the comprehensive intervention package. Sixth, we used complete case analyses which may introduce bias into the results depending on the missing data mechanism.

Several programmatic and policy implications can be drawn from this trial. First, the trial has demonstrated that the essential public health policy, with operational components developed in the design of the trial, is feasible and implementable with minimum input. Though the health gains in the first 12 months are modest due to both practice and policy factors, they may be substantial at the population level when we revisit these indicators as well as CVD events after a longer follow-up period. Policy makers need to consider raising coverage for essential medicines for hypertension and diabetes during for outpatient consultations in primary care facilities, so that patients can be effectively treated in communities and costlier CVD events are prevented. And innovative patient education is clearly needed. There has been strong ownership and involvement of the health authorities, so the comprehensive package will be ready for scale-up if proven successful later.

## Conclusions

The comprehensive intervention package, designed to be replicable within context, was feasible, and implemented as planned by routine health staff. Additional measures, such as more intensive training of doctors, innovative patient education and improved health insurance cover for outpatients are needed. Intervention packages such as this are necessary to ensure quality improvements in primary health facilities regarding CVD risk, hypertension and diabetes care in China and elsewhere.

## Supporting information

S1 Table CONSORT 2010 checklist of information to include when reporting a cluster randomised trial. (PDF)

S1 File Study protocol: Cardiovascular disease risk reduction in rural China: A clustered randomized controlled trial in Zhejiang. Adapted from “Wei X, Zou G, Gong W, Yin J, Yu Y, Walley J, et al. Cardiovascular disease risk reduction in rural China: a clustered randomized controlled trial in Zhejiang. Trials. 2013;14:354”.
(PDF)

S1 Text Intervention strategies to reduce risk of cardiovascular diseases. Adults aged 50–74 years with informed consent, permanent residence in the study area, diagnosed as hypertension with 10-year CVD risk of 20% or higher, or have a recorded medical history of diabetes, in 34 intervention clusters received the intervention package. In 33 control clusters, usual hypertension and diabetes management continued according to their current practice and (if any) knowledge of existing national guidelines.
(DOCX)

S1 Fig Proportion of controlled blood pressure (i.e. systolic blood pressure <140 mmHg and diastolic pressure <90 mmHg) among the 8,178 and 8,443 hypertensive patients recruited in the intervention and control arms respectively, at baseline in 2013/14 and quarterly over 12 months. At quarter 4 there was no significant difference regarding the proportion of patients with hyperten who had their systolic and diastolic blood pressure under control (140/90 mmHg) in the intervention compared with the control arm (OR = 1.07, 95% CI: 0.73 to 1.57, P = 0.74), based on logistic mixed-effects model adjusting for cluster effects, baseline measurements and demographic/socioeconomic factors.
(DOCX)
