# Supplementary material for: Implementation of a comprehensive intervention for patients at high risk of cardiovascular disease in rural China: A pragmatic cluster randomized controlled trial
Source: PLoS One. 2017 Aug 16;12(8):e0183169. doi: 10.1371/journal.pone.0183169 (PMC5559073; doi:10.1371/journal.pone.0183169)
Supplement: S1 Text — Adults aged 50–74 years with informed consent, permanent residence in the study area, diagnosed as hypertension with 10-year CVD risk of 20% or higher, or have a recorded medical history of diabetes, in 34 intervention clusters received the intervention package. In 33 control clusters, usual hypertension and diabetes management continued according to their current practice and (if any) knowledge of existing national guidelines. (DOCX) [file pone.0183169.s003.docx]

| **Target group** | **Theoretical domains** | **Techniques** | **Behavior change modes and content of delivery designed for intervention arm** | **Routine activities/usual care in control arm** |
| --- | --- | --- | --- | --- |
| **Control** |  |  | Usual care in the 33 control cluster township hospital doctors and nurses; practice according to their existing skills and knowledge; i.e. usual hypertension and diabetes management. | **As below** |
| **Intervention** |  | Skill-based training, performance feedback, patient education & treatment support | In the 34 intervention clusters, in addition to their existing knowledge and skills. Intervention package for doctors/nurses includes: operational guidelines, trainings, internal meetings of township hospitals; and for patients and treatment supporters includes: health education messages from doctors/nurses, monthly follow-up appointment and treatment supporter.  The intervention/ training modules were as below: |  |
| Clinical/ public health doctors, nurses | Knowledge Skills Beliefs about capabilities | Information provision | **Operational guidelines** Content: definition of population with high risk of CVD, work flow of patient management, diagnosis methods of hypertension and diabetes, medication strategies and mechanism, lifestyle evaluation and recommendation strategies, strategy of treatment support, management of side effect. **Training of county level at an annual basis and refresher training of township level at a quarterly basis** Content: expended content according to the operational guidelines, consequence of developing CVD, communication skills (using lectures, health education video, case discussions, role play and Q&A). | Training sessions using the China national guidelines of hypertension control and diabetes control are given. But in practice, these guidelines are not frequently referred as they are very thick and not user friendly. No specific training/guidelines on reducing CVD risk and hypertension, diabetes case management. |
|  | Behavior supervision | Monitoring and feedback | **Meetings in township hospitals: performance monitoring** Content: feedbacks of intervention indicators are sent to township hospitals and discussed in the internal meetings of township hospitals monthly, reinforce practice and decide the focus of later refresher training. | Regular internal meetings, however, no specific discussions regarding CVD care. |
| Patient and treatment supporter | Knowledge and beliefs, and need for  treatment support | Information provision | **Health education messages from doctors and nurses during clinic consultation and monthly follow-up** Content: definition and potential consequence of high risk of CVD, benefits of the combined medicines prescribed and recommended healthy lifestyle, potential side effects (using persuasive communication, leaflets, health education video at township hospitals) | Treatment and lifestyle changes are recommended according to existing knowledge and at the individual clinician’s discretion, and based on either hypertension or diabetes instead of holistic approach of CVD risk reduction. |
|  |  | Treatment support | **Monthly follow-up appointment** Content: patients receive follow-ups monthly from doctors/nurses and are reminded of follow-up appointment through phone call/SMS.  **Treatment supporter** Content: doctors/nurses help to designate treatment supporters for their patients and provide trainings on the role of treatment supporter (remind patients of taking medicines, changing lifestyle and time of follow-up appointment) during the consultation. | No specific treatment support. Patients with hypertension or diabetes are followed up once per month/two months/three months based on the disease severity evaluated by doctors. |

S1 Text Intervention strategies to reduce risk of cardiovascular diseases

Adults aged 50-74 years with informed consent, permanent residence in the study area, diagnosed as hypertension with 10-year CVD risk of 20% or higher, or have a recorded medical history of diabetes, in 34 intervention clusters received the intervention package. In 33 control clusters, usual hypertension and diabetes management continued according to their current practice and (if any) knowledge of existing national guidelines.
