# Supplementary material for: Implementation of a comprehensive intervention for patients at high risk of cardiovascular disease in rural China: A pragmatic cluster randomized controlled trial
Source: PLoS One. 2017 Aug 16;12(8):e0183169. doi: 10.1371/journal.pone.0183169 (PMC5559073; doi:10.1371/journal.pone.0183169)
Supplement: S1 Fig — At quarter 4 there was no significant difference regarding the proportion of patients with hyperten who had their systolic and diastolic blood pressure under control (140/90 mmHg) in the intervention compared with the control arm (OR = 1.07, 95% CI: 0.73 to 1.57, P = 0.74), based on logistic mixed-effects model adjusting for cluster effects, baseline measurements and demographic/socioeconomic factors. (DOCX) [file pone.0183169.s004.docx]

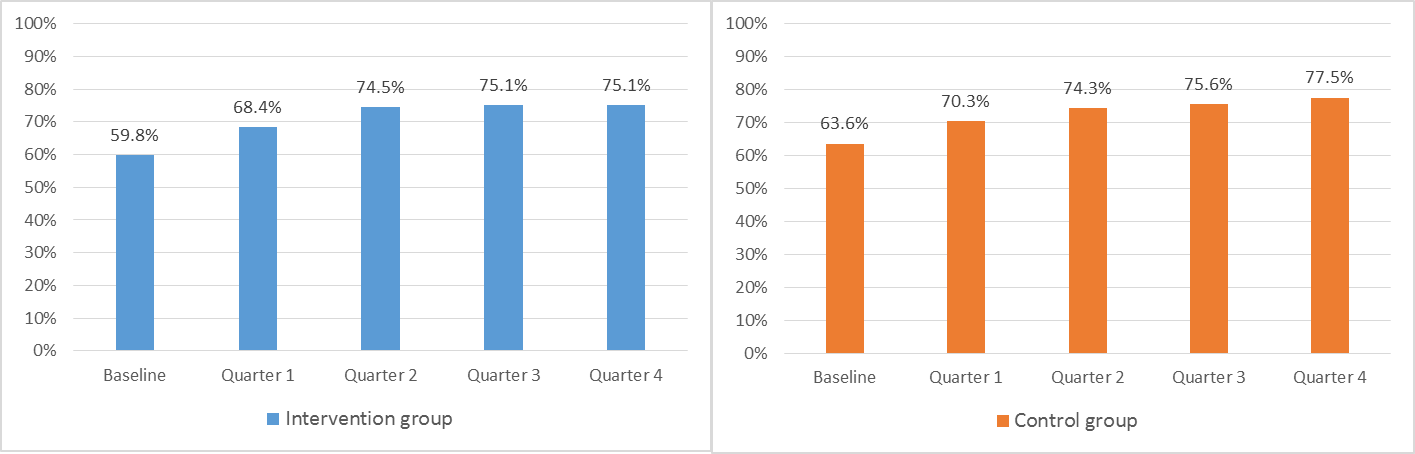


a Proportion of hypertensive patients with blood pressure controlled in the intervention group

b Proportion of hypertensive patients with blood pressure controlled in the control group

S1 Fig Proportion of controlled blood pressure (i.e. systolic blood pressure <140 mmHg and diastolic pressure <90 mmHg) among the 8,178 and 8,443 hypertensive patients recruited in the intervention and control arms respectively, at baseline in 2013/14 and quarterly over 12 months*

* At quarter 4 there was no significant difference in the proportion of hypertensive patients who had under-control blood pressure in the intervention arm compared to the control (OR=1.07, 95% CI: 0.73 to 1.57, P=0.74). Comparison based on logistic mixed-effects model adjusting for cluster effects, baseline measurements and demographic/socioeconomic factors.
